# 

**Published:** 1802-10-01

**Authors:** 


					To CORRESPONDENTS.
Beta's favour is better suited to some of the Theological Reposi-
tories.
ADVERTISE MEN T.
Persons who reside abroad, and who wish to be supplied uith this
Work every month as published, way have it sent to them, FREE OF
POSTAGE, to New York, Halifax, QueVee, and every part of the
West Indus, at three guineas per annum, by Mr. Iiioiixiiill, of
the General Post Office, at No. 21, Sherborne Lane ; to Hamburgh,
Lisbon, Gibraltar, and every part of the Mediterranean, at three
guineas per annum, by Mr. Bishop, of the General Post Office, at
JSo. 22, ShcrborncLane; to the Cape of Good Hope, or to any part
of the East Indies, at two guineas per annum, by Mr. Guv, of the
India House; and to any part of Ireland, at two pounds ten shillings
per annum, by Mr. Smith, of the General Post Office, at No. 3, in
Sherborne Lane. It may also be had of all persons who deal in Books
in every part if the world.
?W. Thorpe, printer, Lici; Court, Fleet Street.^

				

